# Estimation of stature from the foot and its segments in a sub-adult female population of North India

**DOI:** 10.1186/1757-1146-4-24

**Published:** 2011-11-21

**Authors:** Kewal Krishan, Tanuj Kanchan, Neelam Passi

**Affiliations:** 1 Department of Anthropology, Panjab University, Chandigarh-160 014, India; 2 Department of Forensic Medicine and Toxicology, Kasturba Medical College, Mangalore, Manipal University, India

**Keywords:** Forensic podiatry, Personal identification, Anthropometry, Stature estimation, Foot, Sub-adults (adolescents), North-Indian females

## Abstract

**Background:**

Establishing personal identity is one of the main concerns in forensic investigations. Estimation of stature forms a basic domain of the investigation process in unknown and co-mingled human remains in forensic anthropology case work. The objective of the present study was to set up standards for estimation of stature from the foot and its segments in a sub-adult female population.

**Methods:**

The sample for the study constituted 149 young females from the Northern part of India. The participants were aged between 13 and 18 years. Besides stature, seven anthropometric measurements that included length of the foot from each toe (T1, T2, T3, T4, and T5 respectively), foot breadth at ball (BBAL) and foot breadth at heel (BHEL) were measured on both feet in each participant using standard methods and techniques.

**Results:**

The results indicated that statistically significant differences (p < 0.05) between left and right feet occur in both the foot breadth measurements (BBAL and BHEL). Foot length measurements (T1 to T5 lengths) did not show any statistically significant bilateral asymmetry. The correlation between stature and all the foot measurements was found to be positive and statistically significant (*p-value *< 0.001). Linear regression models and multiple regression models were derived for estimation of stature from the measurements of the foot. The present study indicates that anthropometric measurements of foot and its segments are valuable in the estimation of stature. Foot length measurements estimate stature with greater accuracy when compared to foot breadth measurements.

**Conclusions:**

The present study concluded that foot measurements have a strong relationship with stature in the sub-adult female population of North India. Hence, the stature of an individual can be successfully estimated from the foot and its segments using different regression models derived in the study. The regression models derived in the study may be applied successfully for the estimation of stature in sub-adult females, whenever foot remains are brought for forensic examination. Stepwise multiple regression models tend to estimate stature more accurately than linear regression models in female sub-adults.

## Background

Forensic podiatry is the application of sound and researched podiatric knowledge and experience in forensic investigations, to show the association of an individual with a scene of crime, or to answer any other legal question concerned with the foot or footwear that requires knowledge of the functioning foot [1,2]. One of the main tasks of forensic podiatrists is to contribute to the establishment of personal identity in forensic investigations. The need to establish the identity of dismembered remains may arise in cases of mass disasters like terrorist attacks, mass murders, transport accidents, tsunamis, floods, and earthquakes. Estimation of stature is an important parameter in forensic investigation and is considered as one of the 'big fours' of forensic anthropology. Stature, age, sex and ancestry facilitate the narrowing down of the pool of possible victim matches in the forensic investigation process and help in establishing identification of the individual. Stature can be estimated from skeletal remains and body parts owing to the established relationship between stature and different parts of the body [3-7].

Forensic identification from the foot and its parts is important as there is an increased likelihood of the recovery of feet (often enclosed in shoes), separated from the body, in mass disasters such as high power explosions and bomb blasts, air plane crashes and other high impact transportation accidents [8]. The significance of the human foot and its bones, and foot prints in identification has been successfully reported in the past [2]. Published literature on estimation of sex from foot bones and foot dimensions [9-13], individualistic and unique features of the foot and footprints [13-18], and the use of radiographic comparisons of the foot [19-23] confirms the importance of the foot in identification. Kanchan et al [24] have reported the correlation of hand and foot dimensions for personal identification in mass disasters. Earlier studies of the estimation of stature from human foot bones [25-29], foot dimensions [30-39], foot prints and foot outline [40-42] reveal that the human foot, its bones and its impressions can successfully be used in estimation of stature in forensic and legal examinations.

Stature estimation is commonly reported in forensic case work pertaining to adult populations and less commonly in sub-adult cases [43]. Even the earlier studies on estimation of stature from foot measurements were conducted on adult populations [30-39]. Studies to establish standards for stature estimation in a sub-adult population are essential as the formula derived for stature estimation in the adult population cannot be applied to sub-adults. In the case of growing individuals, it is probably more useful to estimate age than stature. Once the age is established, estimation of stature can reduce the pool of possible victim matches even further. The present study on the estimation of stature from the foot and its segments was thus conducted on a sub-adult female population. A detailed anthropometric analysis of seven foot measurements was conducted in the study sample. The purpose of the present study was to correlate stature with various anthropometric measurements of the foot and its segments and estimate stature from these measurements using linear and multiple regression models in a sub-adult female population of North India. The study is intended to formulate standards for the estimation of stature from the foot and its parts in a sub-adult female population of North India.

## Methods

**Figure 1:**
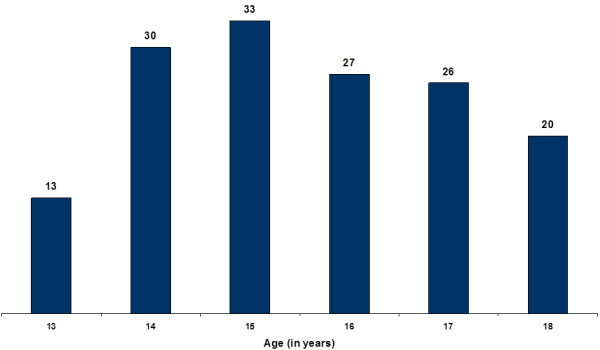
**Age distribution of the study sample**.

The study was conducted in a selected area of Tehsil Kalka, in the District of Panchkula in Haryana state, Northern India. The data were collected on a sample of 149 North Indian sub-adult females. The participants were aged 13 to 18 years (Mean age 15.5 ± 1.6 years). Age distribution for the study sample is shown in Figure 1. Healthy individuals were included in the study after taking informed consent. The data were collected in the month of October-November 2006 from the educational institutions located in the villages of Nanakpur, Marranwala and Bassolan. The participants were taken from a mixed population of the area i.e. belonging to caste groups *Lobana, Saini, Gujjar, Kumhar, Teli, Nai, Dhiman *and *Lohar*. These are the major caste groups of North India, strictly marrying within their own caste. The majority of the individuals from these caste groups are engaged in agriculture, and animal husbandry.

### Data collection and anthropometry

The data for the present study included stature, length of the foot from each toe (T1, T2, T3, T4, and T5 respectively), foot breadth at the ball and foot breadth at the heel. All the measurements were taken with standard procedures and landmarks defined by Krishan [8], Robbins [14] and Vallois [44]. The following techniques were used while taking measurements:

**Stature**: Stature is the vertical distance between the point vertex (highest point on the head when the head is held in the Frankfurt Horizontal plane) and the floor. Each participant was asked to stand up against the wall with hands hanging down, feet axes parallel or slightly divergent, and head in the Frankfurt Horizontal plane. Thus, the participant was made to stand in an erect posture without any headgear or footwear being worn and stature was recorded using an anthropometer. No pressure was exerted since this is a contact measurement.

**Figure 2:**
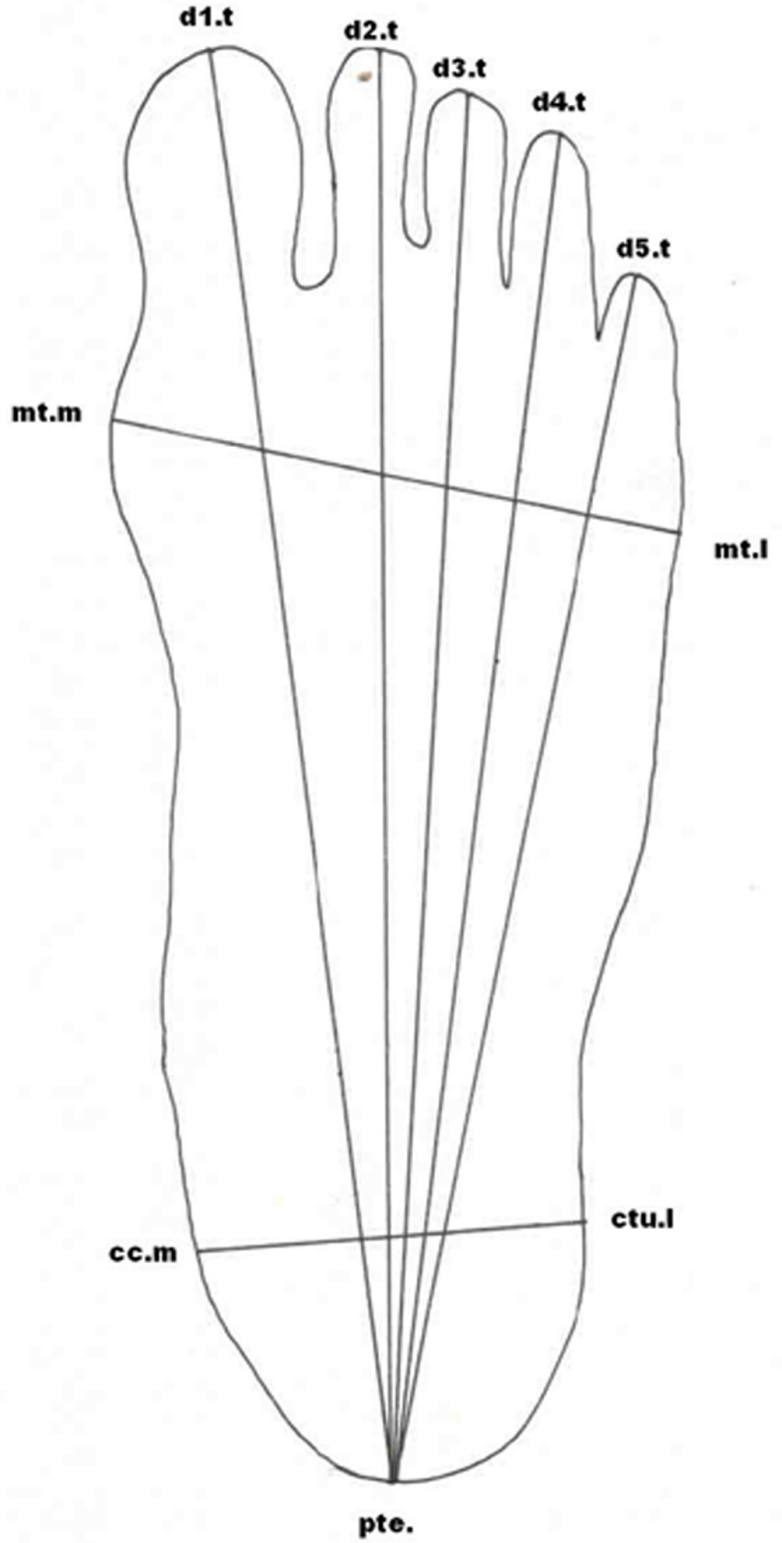
**Measurements and landmarks on the human foot**.

For recording foot measurements, the participant was made to stand so that both feet were slightly apart with equal pressure on both. The sliding caliper was placed horizontally on the landmarks and the measurement was taken. All the anthropometric measurements taken on the foot are depicted in Figure 2. Different landmarks on the foot are described in Table 1.

**Table 1:** Landmarks used in foot measurements, and their description

Landmark	Description
Pternion (pte)	Most projected point on the back of the heel when the participant is standing

Digit 1, terminal (d1.t)	Most anterior point on the terminal phalanx of the first toe

Digit 2, terminal (d2.t)	Most anterior point on the terminal phalanx of the second toe

Digit 3, terminal (d3.t)	Most anterior point on the terminal phalanx of the third toe

Digit 4, terminal (d4.t)	Most anterior point on the terminal phalanx of the fourth toe

Digit 5, terminal (d5.t)	Most anterior point on the terminal phalanx of the fifth toe

Metatarsal medial (mt.m)	Joint of the anterior epiphyses of the first metatarsal

Metatarsal lateral (mt.l)	Joint of the anterior epiphyses of the fifth metatarsal

Calcaneal tubercle lateral (ctu.l)	Most lateral side of the heel

Calcaneal concavity medial (cc.m)	Most medial side of the heel

**T1 Length (d1.t-pte.): **Distance from pternion (pte) to the most distal part of the first toe (d1.t).

**T2 Length (d2.t-pte.): **Distance from pternion (pte) to the most distal part of the second toe (d2.t).

**T3 Length (d3.t-pte.): **Distance from pternion (pte) to the most distal part of the third toe (d3.t).

**T4 Length (d4.t-pte.)**: Distance from pternion (pte) to the most distal part of the fourth toe (d4.t).

**T5 Length (d5.t-pte.)**: Distance from pternion (pte) to the most distal part of the fifth toe (d5.t).

Pternion (pte.) is the most posteriorly projecting point on the heel when the participant stands erect.

**Foot breadth at ball (mt.l-mt.m): **Distance between the joint of the anterior epiphyses of the first metatarsal (mt.m), the most prominent part of the inner side of the ball of the foot, and the joint of the anterior epiphyses of the fifth metatarsal (mt.l), the most prominent part of the outer side of the ball of the foot.

**Foot breadth at heel (cc.m-ctu.l): **Distance taken from the lateral side of the heel (ctu.l) to the medial side (cc.m) of the heel.

All the measurements were taken with the help of standard instruments; anthropometric rod and sliding caliper. The information about the age of the participants was taken from school records and was cross-checked with the participants. Measurements were recorded to the nearest millimeter. Only healthy participants free from any deformity of the foot were included in the study.

### Technical/Measurement error

While conducting the present study, the technical error of the measurement and intra-observer error inherent in anthropometry were taken into consideration. While collecting data in the field, the instruments were regularly checked for their accuracy. All the measurements were taken by one individual, trained physical anthropologist (NP), to avoid inter-personal or inter-observer error. Before beginning data collection, all measurements were taken on 15 participants twice and the technical error calculated following Schell et al. [45]. The measurement error is defined as the square root of the sum of the squared deviations divided by twice the sample size (S^2 ^= √Σd^2^/2n). The same formula was applied to the left-right foot differences. The value of 'F' statistics (F-ratio) was calculated. Table 2 presents the sizes of the technical error variance of the foot measurements. The technical error within the measurements taken from each foot (left or right) and between each of these feet is described as the 'S2 within foot' and 'S2 between feet" and the ratio of the two errors is distributed as an F-statistic. The F-ratio for all the measurements is statistically significant at the α-level of P < 0.01. Hence, the variation between left and right feet respectively is several times larger than the measurement error, indicating that measurement error contributed little to the apparent difference between left and right feet. The findings indicate that the measurements are reproducible and without significant technical error.

**Table 2:** Technical error of measurement within and between sides of foot dimensions

Foot dimension	S^2 ^(Within side)	S^2 ^(Between side)	F-ratio
T1	0.0527	0.3866	7.3350*

T2	0.0321	0.2242	6.9844*

T3	0.0436	0.2687	6.1628*

T4	0.0429	0.1987	4.6317*

T5	0.0178	0.1873	10.5224*

BBAL	0.0499	0.1816	3.6392*

BHEL	0.0291	0.4153	14.2714*

T1-T1 Length (d1.t-pte), T2-T2 Length (d2.t-pte), T3-T3 Length (d3.t-pte), T4-T4 Length (d4.t-pte), T5-T5 Length (d5.t-pte), BBAL-Foot breadth at ball (mt.m-mt.l), BHEL-Foot breadth at heel (cc.m-ctu.l) *p < 0.01

### Data analysis

The data were statistically analyzed using SPSS (Statistical Package for Social Sciences, version 11.0) computer software (SPSS, Inc., Chicago, IL, USA). Asymmetry between sides in the foot measurements was calculated and tested using a paired t-test [45]. Pearson's correlation coefficients were calculated to find the correlation between stature and various measurements of the foot. Stature was estimated from the foot and its various measurements by using linear and multiple regression analysis. A *p-value *of less than 0.05 was considered to be significant. The linear regression models for stature estimation are derived as S (stature) = a + b x ± SEE, where, 'a' is constant, 'b' is the regression coefficient of the independent variable i.e. individual foot measurement, 'x' is an individual variable/foot measurement and SEE is Standard Error of Estimate. Multiple regression models were calculated for reconstruction of stature from foot length (T1 to T5) and foot breadth measurements. Step-wise multiple regression models were derived as S (stature) = a (constant) + b1 (1: regression coefficient of the variable) × X1 (1: variable) + b2 (2: regression coefficient of the variable) × X2 (2: variable) +... bn (n: regression coefficient of the variable) × Xn (n: variable) ± SEE.

## Results and discussion

Descriptive statistics of foot dimensions (cm) on the right and left sides and the side differences (right-left) are shown in Table 3. Statistically significant (p < 0.05) side differences occurred in the foot breadth measurements (BBAL and BHEL). Foot length measurements (T1 to T5 lengths) did not show any statistically significant bilateral asymmetry between left and right feet (p > 0.05).

**Table 3:** Descriptive statistics of foot dimensions (cm) on right and left sides and side (Right-Left) differences

	Right foot			Left foot			t-value	p-value
	**Range**	**Mean**	**S.D**.	**Range**	**Mean**	**S.D**.		

T1	20.7-27.7	23.2	1.1	20.6-25.7	23.2	1.0	0.213	0.832

T2	19.7-24.6	22.7	1.0	19.9-24.9	22.7	0.9	-0.769	0.443

T3	19.1-24.0	21.8	1.0	19.0-24.0	21.8	0.9	-0.562	0.575

T4	18.1-23.0	20.6	0.9	18.0-23.0	20.5	0.9	0.309	0.758

T5	16.5-21.3	19.1	0.9	16.6-21.4	19.1	0.9	-0.734	0.464

BBAL	7.6-10.1	8.9	0.5	7.7-10.3	9.1	0.5	-4.554	< 0.001

BHEL	5.1-6.7	5.9	0.4	4.9-6.8	5.7	0.4	5.586	< 0.001

S.D.-standard deviation, T1-T1 Length (d1.t-pte), T2-T2 Length (d2.t-pte), T3-T3 Length (d3.t-pte), T4-T4 Length (d4.t-pte), T5-T5 Length (d5.t-pte), BBAL-Foot breadth at ball (mt.m-mt.l), BHEL-Foot breadth at heel (cc.m-ctu.l)

Table 4 shows the descriptive statistics for stature and foot measurements in sub-adult females across different ages. The table indicates the variations observed in foot measurements and stature through the different ages in the study sample. Mean stature and foot measurements did not show significant variation through the different ages (p > 0.05). In the age 14 years group, an outlying stature value of 183.9 cm is observed that has probably resulted in the higher standard deviation SD for stature in this group.

**Table 4:** Descriptive statistics for age distribution (years) of stature and foot measurements (cm)

	Age	13 (n = 13)	14 (n = 30)	15 (n = 33)	16 (n = 27)	17 (n = 26)	18 (n = 20)
Variable							

T1	Mean (SD)	22.9 (1.2)	23.1 (0.9)	23.0 (1.0)	23.4 (1.2)	23.2 (0.9)	23.6 (0.9)

	Range	21.3-24.9	21.4-25.7	21.0-25.5	20.7-25.6	21.6-24.9	21.7-24.9

T2	Mean (SD)	22.5 (1.0)	22.5 (0.8)	22.5 (0.9)	22.8 (1.1)	22.9 (0.9)	23.0 (0.9)

	Range	21.3-24.7	20.6-24.1	20.5-24.7	19.8-24.6	21.3-24.6	21.1-24.7

T3	Mean (SD)	21.7 (0.9)	21.7 (0.8)	21.5 (1.0)	21.8 (1.1)	22.0 (0.9)	22.1 (0.8)

	Range	20.6-23.6	20.0-22.9	19.2-24.0	19.0-23.7	20.4-23.4	20.5-23.6

T4	Mean (SD)	20.4 (0.9)	20.5 (0.8)	20.3 (0.9)	20.6 (1.0)	20.8 (0.8)	20.9 (0.8)

	Range	19.1-22.4	19.0-22.5	18.5-23.0	18.1-22.4	19.2-22.2	19.3-22.8

T5	Mean (SD)	19.0 (0.9)	18.9 (0.6)	18.9 (0.9)	19.1 (1.2)	19.4 (0.8)	19.5 (0.6)

	Range	17.8-20.5	17.9-20.6	17.1-21.3	16.7-21.4	17.9-20.5	17.8-20.9

RBBAL	Mean (SD)	8.7 (0.7)	8.9 (0.4)	8.9 (0.5)	9.1 (0.5)	8.9 (0.4)	9.1 (0.5)

	Range	7.8-10.0	8.3-10.0	7.7-9.8	8.2-10.1	7.6-9.7	8.0-9.8

LBBAL	Mean (SD)	8.9 (0.5)	8.9 (0.3)	9.0 (0.5)	9.2 (0.5)	8.9 (0.4)	9.1 (0.5)

	Range	8.0-10.0	8.4-9.8	7.8-10.1	8.3-10.3	7.7-9.5	8.1-10.2

RBHEL	Mean (SD)	5.8 (0.5)	5.7 (0.3)	5.8 (0.4)	5.9 (0.4)	5.9 (0.3)	6.0 (0.4)

	Range	5.2-6.7	5.1-6.2	5.1-6.5	5.2-6.7	5.3-6.4	5.4-6.6

LBHEL	Mean (SD)	5.7 (0.5)	5.6 (0.3)	5.8 (0.4)	5.9 (0.4)	5.8 (0.3)	5.9 (0.4)

	Range	4.9-6.5	5.2-6.3	5.1-6.5	5.1-6.8	4.9-6.4	5.2-6.7

Stature	Mean (SD)	152.1 (5.8)	153.2 (7.3)	153.1 (5.4)	155.6 (6.4)	154.8 (3.5)	157.0 (5.2)

	Range	143.8-162.1	142.3-183.9	141.7-163.9	141.9-164.7	147.9-161.0	149.4-166.0

S.D.-standard deviation, T1-T1 Length (d1.t-pte), T2-T2 Length (d2.t-pte), T3-T3 Length (d3.t-pte), T4-T4 Length (d4.t-pte), T5-T5 Length (d5.t-pte), BBAL-Foot breadth at ball (mt.m-mt.l), BHEL-Foot breadth at heel (cc.m-ctu.l)

Pearson's correlation coefficients (r) between stature and various foot measurements on the right and left sides are shown in Table 5. All the correlation coefficients were found to be statistically significant (p < 0.001). Thus, stature is positively and significantly related to various foot measurements. Foot length measurements however, show higher correlation coefficients than foot breadth measurements.

**Table 5:** Pearson Correlation (r) between stature and different foot dimensions

Foot Dimension	Right	Left
T1	0.581*	0.661*

T2	0.589*	0.583*

T3	0.554*	0.601*

T4	0.521*	0.577*

T5	0.570*	0.616*

BBAL	0.353*	0.375*

BHEL	0.405*	0.376*

T1-T1 Length (d1.t-pte), T2-T2 Length (d2.t-pte), T3-T3 Length (d3.t-pte), T4-T4 Length (d4.t-pte), T5-T5 Length (d5.t-pte), BBAL-Foot breadth at ball (mt.m-mt.l), BHEL-Foot breadth at heel (cc.m-ctu.l), *p-value < 0.001

Foot length measurements (T1 to T5) did not show any differences between left and right feet, hence the mean of right and left feet together was used to derive linear regression models from individual foot lengths from T1 to T5 (Table 6). However, for stature estimation from the foot breadth measurements (BBAL, BHEL), linear regression models were derived for right and left feet individually (Table 7). These regression models may be applied in stature estimation from the foot and its various segments independently. It is observed that stature can be estimated more accurately from foot length measurements than foot breadth measurements. Among the foot measurements, T1 gives the most accurate estimation of stature by linear regression analysis. Although the SEE value is minimal and the predictive accuracy (R^2^) maximum for T1, accuracy of all measurements in stature estimation were comparable (Table 6). The accuracy of foot breadth measurements in stature estimation is less than that of the foot length measurements.

**Table 6:** Linear regression models for reconstruction of stature from foot length measurements.

Variable	Regression model	S.E.E (cm)	R	R^2^	p-value
T1	69.346 + 3.663 (T1)	4.568	0.636	0.405	< 0.001

T2	70.679 + 3.688 (T2)	4.745	0.598	0.358	< 0.001

T3	75.341 + 3.630 (T3)	4.798	0.586	0.343	< 0.001

T4	79.009 + 3.665 (T4)	4.921	0.556	0.309	< 0.001

T5	76.106 + 4.090 (T5)	4.716	0.605	0.366	< 0.001

T1-T1 Length (d1.t-pte), T2-T2 Length (d2.t-pte), T3-T3 Length (d3.t-pte), T4-T4 Length (d4.t-pte), T5-T5 Length (d5.t-pte), S.E.E-Standard Error of Estimate

**Table 7:** Linear regression models for reconstruction of stature from foot breadth measurements.

Variable	Regression model	S.E.E (cm)	R	R^2^	p-value
RBBAL	115.149 + 4.369 (RBBAL)	5.540	0.353	0.125	< 0.001

LBBAL	112.483 + 4.619 (LBBAL)	5.488	0.375	0.141	< 0.001

RBHEL	114.645 + 6.761 (RBHEL)	5.413	0.405	0.164	< 0.001

LBHEL	121.312 + 5.721 (LBHEL)	5.488	0.376	0.141	< 0.001

RBBAL-Right Foot breadth at ball (mt.m-mt.l), RBHEL-Right Foot breadth at heel (cc.m-ctu.l), LBBAL-Left Foot breadth at ball (mt.m-mt.l), LBHEL-Left Foot breadth at heel (cc.m-ctu.l), S.E.E-Standard Error of Estimate

Stepwise multiple regression models are derived for estimation of stature from foot length (T1 to T5) and foot breadth measurements (BHEL and BBAL) as shown in Table 8 and Table 9 respectively. Since bilateral asymmetry exists in foot breadth measurements, multiple regression models are derived on both left and right sides separately. Multiple regression models derived from the measurement of the foot length (T1 to T5) estimate stature more accurately than models derived from the measurements of the foot breadth (BHEL and BBAL). It is further observed that the multiple regression models tend to estimate stature more accurately than the respective linear regression models for length and breadth measurements.

**Table 8:** Step-wise regression models for reconstruction of stature from foot length (T1 to T5) measurements

Variable	Regression model	S.E.E (cm)	R	R^2^
T1 to T5	67.535 + 2.574(T1*) + 0.102(T2) + 2.115(T3)-3.356(T4*) + 2.495(T5*)	4.516	0.659	0.434

T1-T1 Length (d1.t-pte), T2-T2 Length (d2.t-pte), T3-T3 Length (d3.t-pte), T4-T4 Length (d4.t-pte), T5-T5 Length (d5.t-pte), *p-value < 0.05, S.E.E-Standard Error of Estimate

**Table 9:** Step-wise regression models for reconstruction of stature from foot breadth (BBAL and BHEL) measurements on right and left side

Variable	Regression model	S.E.E (cm)	R	R^2^
RBBAL, RBHEL	108.164 + 1.793(RBBAL) + 5.126(RBHEL*)	5.394	0.419	0.176

LBBAL, LBHEL	108.849 + 2.803(LBBAL*) + 3.481(LBHEL*)	5.409	0.414	0.171

RBBAL-Right Foot breadth at ball (mt.m-mt.l), RBHEL-Right Foot breadth at heel (cc.m-ctu.l), LBBAL-Left Foot breadth at ball (mt.m-mt.l), LBHEL-Left Foot breadth at heel (cc.m-ctu.l), *p-value < 0.05, S.E.E-Standard Error of Estimate

Age and growth velocity are important factors to be considered in correlating foot measurements and stature. In sub-adults, foot measurements are naturally correlated with age but the phenomenon is complicated by differences in rates of growth between the individuals [46,47]. The average adult length of foot is attained by the age of 16 years in males and 14 years in females [48,49]. During this period, there is a growth spurt and long bones continue to grow leading to an increase in stature untill the individual attains maturity. Stature estimation in the case of sub-adults is even more difficult because of the ongoing physical growth of the thorax region and long bones of the lower limbs which contribute substantially to stature of an individual. In the past, stature estimation studies have been largely conducted on the adult population primarily owing to adolescent growth spurt and the effect of growth on long bones of the body. Very obviously the formula derived for the adult population cannot be applied to sub-adults. Keeping in view the lack of systematic studies on stature estimation from foot measurements in adolescents or the sub-adult population, and in the wake of rising incidence of teenage crimes in India [50], the study may be helpful in the estimation of stature where other possible means of identification are not useful. To the best of our knowledge no similar studies have been conducted on stature estimation in a sub-adult population and hence the findings of the study cannot be compared per se.

In such a forensic investigation, inter-observer and intra-observer error play an important role in the accuracy and reproducibility of the anthropometric measurements. In the present study, utmost care was taken to ensure the precision of anthropometric measurements. The measurement errors have a substantial effect on the accuracy and reliability of the standards in forensic science which ultimately affect the forensic anthropology case work involving anthropometry [51]. Our study reveals no significant variation and errors associated with the technique in anthropometric measurements (Table 2). Therefore, a set of standards in the estimation of stature from foot and its parts produced by the present study are reliable.

## Conclusions

The present study concludes that foot measurements have a strong relationship with stature in the sub-adult female population of North India. Hence, the stature of an individual can be successfully estimated from the foot and its segments using different regression models derived in the study. It was observed that the regression models derived from foot length measurements were more reliable than those from foot breadth measurements in the prediction of stature in forensic examinations. Stepwise multiple regression models tend to estimate stature more accurately than linear regression models in female sub-adults. Similar studies on a male sub-adult population are proposed. It is highlighted here that the findings of the present research apply to a very specific population (the sub-adult female population of North India) and hence, should not be generalised. Researchers are encouraged to conduct similar studies in different population groups to look into the generation of additional standards which can further be used in the identification of individuals from human remains.

## Competing interests

The authors declare that they have no competing interests.

## Authors' contributions

KK conceived, designed contributed to the introduction, material and methods and discussion, wrote, reviewed and edited the manuscript. TK analyzed data, made all the tables and figures, wrote results and also contributed to introduction and discussion. NP collected all the data and entered data on the computer and helped in the review of literature. All authors have read and approved the final manuscript.

## Authors' information

Kewal Krishan^1^, MSc, PhD, serving as Senior Assistant Professor at Department of Anthropology (UGC Centre for Advanced Studies and FIST Department), Panjab University, Chandigarh, India. Tanuj Kanchan^2 ^MD, serving as Associate Professor, Department of Forensic Medicine and Toxicology, Kasturba Medical College, Mangalore, (Affiliated to Manipal University), India.

Neelam Passi^13^MSc, University Grants Commission Rajiv Gandhi National Fellow at Department of Anthropology (UGC Centre for Advanced Studies and FIST Department), Panjab University, Chandigarh, India.
